# Integration of work, environment, and human health - agendas for sustainability and decent work

**DOI:** 10.47626/1679-4435-2025-1423

**Published:** 2025-07-08

**Authors:** Rafael Buralli, Kelly Polido Kaneshiro Olympio, Armando Meyer, Maria Teresa Muñoz-Quezada

**Affiliations:** 1 Departamento de Política, Gestão e Saúde, Faculdade de Saúde Pública, Universidade de São Paulo (USP), São Paulo, SP, Brazil; 2 Grupo de Pesquisa Expossoma e Saúde do Trabalhador, Departamento de Saúde Ambiental, Faculdade de Saúde Pública, USP, São Paulo, SP, Brazil; 3 Programa de Saúde Ambiental e Ocupacional, Instituto de Saúde Pública, Universidade Federal do Rio de Janeiro, Rio de Janeiro, RJ, Brazil; 4 Escuela de Salud Pública, Facultad de Medicina, Universidad de Chile, Santiago, Chile

**Keywords:** occupational health, environmental health, health policy, health surveillance., saúde ocupacional, saúde ambiental, política de saúde, vigilância em saúde pública.

## Abstract

Recent environmental and work-related changes (and deterioration) have negatively affected the population, exacerbating conflicts and health issues. This essay reflects on how work and the environment are intrinsically connected as determinants, which can be sources of both health and illness, and discusses possible agendas and pathways for advancing sustainability and decent work. While several international and national strategies and policies acknowledge the right to a healthy environment and decent work (and the interactions between these essential factors for life), the implementation of these agendas remains a major challenge, especially in the context of the climate crisis. In addition to recognizing the inseparable relationship between work, environment, and health and promoting integrated policies and actions to achieve environmental justice and decent work, it is urgent to establish more transversal strategies grounded in the specific realities and needs of different territories, prioritize the most vulnerable populations and workers, strengthen the monitoring and control of environmental and occupational hazards and exposures, ensure spaces for participation, and provide workers and society with training for active and qualified engagement.

## Abstract (pt)

As recentes transformações e deteriorações ambientais e do mundo do trabalho têm afetado negativamente a população, amplificando os conflitos e os agravos à saúde. Este ensaio reflete sobre como o trabalho e o ambiente são determinantes intrinsecamente conectados, que podem ser fontes de saúde ou adoecimento, e discute algumas agendas e caminhos possíveis para o avanço da sustentabilidade e do trabalho decente. Ainda que muitas estratégias e políticas internacionais e nacionais reconheçam o direito ao ambiente saudável e trabalho decente, sendo as interações entre esses fatores essenciais à vida, a implementação dessas agendas ainda é um enorme desafio, especialmente no contexto de crise climática. Além de reconhecer a relação indissociável entre trabalho, ambiente e saúde, e promover políticas e ações integradas para alcançar a justiça ambiental e o trabalho decente, é urgente estabelecer estratégias mais transversais e ancoradas na realidade e nas necessidades dos territórios, priorizar populações e trabalhadores mais vulneráveis, reforçar a fiscalização e o controle de riscos e exposições ambientais e ocupacionais, garantir espaços de participação e capacitar trabalhadores e sociedade para uma participação ativa e qualificada.

## INTRODUCTION

Work and environment can be sources of both health and illness. Positive environmental and working conditions can promote healthy habits and support an active, dignified, and rewarding life. Decent and meaningful work has profound implications for individuals, organizations, and society. Access to decent work, with better-paying jobs aligned with professional skills and expectations, can improve physical and mental health, income, and well-being for individuals and their families.^1^

Healthy and sustainable environments, with readily available and high-quality natural resources and food, can result in good health conditions and promote individual prosperity. For example, living near green spaces has been increasingly associated with positive impacts on both physical and mental health, the development of strong social relationships, and the adoption of healthy lifestyle choices, such as physical activity. In addition, the preservation of these environments is essential in tackling climate change.^2^

The workplace can influence workers’ physical activity levels and reduce sedentary behavior. Measures that can be implemented in the workplace include providing sit-stand desks, encouraging stretching and physical activity breaks, creating dedicated spaces for physical activity, supporting active commuting such as cycling or walking, and promoting the use of stairs for those without mobility limitation.^3,4^

Despite the available scientific knowledge, recent environmental and work-related changes - and deterioration - have negatively affected the health and well-being of communities, exacerbating conflicts, health issues, and their determinants. Unemployment, new employment models, informality, outsourcing services to third-party providers - and often to fourth-party providers -, payment on a piecework basis, and the “uberization” of work are contributing to illness as well as to precarious employment relationships and living and working conditions for thousands of workers annually.^5,6^ Regardless of the nature of the employment relationship, whether formal or informal, all workers have the right to decent, inclusive, healthy, and sustainable work, along with a social protection network and access to basic services for a dignified livelihood.^7^

Work can generate and perpetuate environmental and health inequalities. Production models that lack environmental sustainability increase the pressure on natural resources and heighten the risk of disasters and occupational accidents, threatening the lives and health of both workers and the general population. Examples include the collapses of the Vale and Samarco mining tailings dams in Minas Gerais, Brazil. These disasters released millions of tons of mineral waste and metals, causing unprecedented impacts on people’s lives, the environment, and resource availability. The resulting water, air, and soil contamination, shifts in disease vectors, and infrastructure damage can lead to numerous acute and chronic diseases.^8^

The current trends of globalization, technological advances, and automation demand adjustments at both individual and collective levels. While these situations can restructure production processes to increase profits for companies and investors, they may also compromise the physical and mental health of workers. These workers often lack the appropriate support to adapt to new technologies and processes, acquire new skills, and cope with continuous changes in their work environments and processes. Without proper training and the provision of these supportive conditions, such changes can pose risks to workers’ health.^7^ Conversely, new technologies also offer opportunities, especially for younger workers, in terms of facilitating training, fostering entrepreneurship, creating new jobs, increasing productivity, and strengthening the capacity of governments to implement public policies.^9^

The quality of the natural and built environment, coupled with increasingly common environmental disasters, influence both the availability and quality of work, as well as individuals’ income. Recent changes in the natural environment are largely a consequence of pressure exerted by human activity on the environment and its resources, accelerated by unsustainable land use and production processes. According to Frank et al.,^10^ there are other emerging socio-environmental challenges that have a direct relationship with work and health, including:

### INFLUENCE OF TECHNOLOGY ON THE NATURE OF WORK

The COVID-19 pandemic has accelerated the adoption of new technologies, contributing to a rise in precarious work and income instability. Many workers have transitioned to remote work, often without due attention to their home environmental and working conditions, which include factors such as ergonomics, lighting, noise levels, air quality, length of the working day, food security, and risks of social isolation.^10,11^

### WORKPLACE DISCRIMINATION AND ENVIRONMENTAL RACISM

Systemic disparities affect both working and environmental conditions, influenced by gender, race, ethnicity, and age. Men commonly hold higher-paying management positions, while women predominate in care and administrative positions. Historically, racial minorities, such as Black, Mixed-race, Indigenous, and *quilombola* communities, have faced marginalization and lived in areas with limited access to essential services, in addition to holding a larger share of high-risk occupations.^10^ During the COVID-19 pandemic, these marginalized groups experienced disproportionately higher rates of hospitalization and death, especially among non-White communities in Brazil^12^ and the United States.^13^ Vulnerable populations, including delivery and domestic workers, faced greater exposure to the virus, highlighting the risks associated with proximity to air pollutants and precarious working conditions,^14^ underscoring the urgent need to address such inequities.

### MIGRATION AND WORK

Refugees and migrant workers often endure suffering, trauma, and health issues as they are compelled to abandon their lives, jobs, and careers in their home countries in pursuit of new opportunities and a dignified life elsewhere. They frequently find themselves in situations of accepting precarious contracts, temporary and insecure employment, and work that is physically and psychologically demanding and dangerous.^10^ Environmental disasters force millions of people to move within or across borders in search of safety and better living conditions. The number of environmental and climate refugees is growing rapidly worldwide. While often overlooked, they join those fleeing war and conflict, putting pressure on health systems and exacerbating geopolitical, social, and environmental crises, particularly in lower-income countries.^15^

### CLIMATE CRISIS

The planet is warming rapidly and uncontrollably. Extreme weather events are becoming increasingly common, causing significant impacts and establishing climate change as the most serious environmental crisis of our era.^16,17^ Disasters such as heatwaves, floods, landslides, droughts, wildfires, storms, and hurricanes, all resulting from climate change, can harm the health of workers and communities, exacerbating social and health inequalities.^10,17^

These events affect physical and mental health, contributing to suffering, acute and chronic illnesses, mental health disorders, malnutrition, allergies, cardiovascular, respiratory, renal, and infectious diseases, poisoning, accidents, injuries, waterborne diseases, zoonoses, and vector-borne diseases.^10,17-19^ Currently, approximately 3.6 billion people live in areas highly vulnerable to the effects of climate change. Projections indicate that between 2030 and 2050, the climate crisis will result in an estimated 250,000 additional deaths per year from undernutrition, malaria, diarrhea, and heat stress.^17^

The effects of climate change, which are already affecting and will continue to increasingly affect workers globally, highlight the connections between the environment, work, and health. These impacts include rising temperatures, air pollution, exposure to ultraviolet radiation, extreme weather conditions, vector-borne diseases, and changes in industries and the built environment.^19^ The effects on workers’ health can be direct, such as floods and landslides that can devastate communities and local economies, and droughts that can impact agriculture; or indirect, by deteriorating water and air quality, compromising food availability and quality, and causing changes in land use and ecological disturbances, which can increase the incidence of waterborne, vector-borne, or air pollution-related diseases. While climate change impacts the entire population, the most vulnerable individuals are disproportionately more affected.^10,20^

The environmental and economic impacts of climate change on people’s health have been widely discussed. However, when it comes to worker’s health, international reports often focus only on the economic consequences for labor productivity, overlooking the direct effects on workers’ health.^16,21^ Scientific research commonly focuses on the health risks associated with extreme heat exposure in sectors such as agriculture and construction, neglecting a wide range of climate change-related hazards that affect many other categories of workers,^22^ such as street vendors, urban cleaning workers, recyclable waste collectors, cooks, fishermen, and shellfish gatherers. Essential workers who are heavily impacted by these changes, but often overlooked in public policy and academic discussions, also include health care professionals, firefighters, rescue teams, civil defense personnel, and both hired and volunteer wildland firefighters, all of whom are on the front lines of emergency and disaster preparedness and response.

### POLITICAL AGENDAS AND POSSIBLE PATHWAYS

Decent work is recognized as a human right by the Universal Declaration of Human Rights, and a safe and healthy workplace is considered a fundamental right by the International Labour Organization. Both highlight the importance of access to decent and safe work, characterized by fair and favorable conditions, protection against unemployment, equal pay, social protection, and the right to join labor unions and participate in organizations.^5^ This right is further supported by several international conventions aimed at eliminating workplace discrimination based on race and gender, protecting children’s rights, and safeguarding migrant workers and their families. However, major challenges persist, and clear guidelines are needed to ensure decent, safe, and healthy work environments and processes for all workers, regardless of the nature of their employment relationship.

International agendas outline urgent directions and actions, such as the United Nations’ 2030 Agenda for Sustainable Development, also known as the Sustainable Development Goals (SDGs).^23^ Rooted in sustainability, this agenda acknowledges the interconnectedness of global issues and solutions, highlighting the influence of social and environmental determinants on individuals’ health conditions. Therefore, it is essential to identify and confront inequalities that disproportionately impact the most vulnerable populations - those with limited access to essential services, precarious living and working conditions, and who experience the unequal distribution of resources, income, goods, services, and products.^24^

SDGs are intrinsically connected and deeply related to workers’ health, indicating pathways and offering contributions to the achievement of the 2030 Agenda. Key areas where this impact is evident include the effective management of risks and resources, access to sustainable environments and safe sanitation, the eradication of chronic, communicable, and neglected diseases and poisonings, and the implementation of more robust fiscal, wage, and social protection policies.^24,25^ Specifically, some goals and targets that directly affect workers’ health include:

Health and well-being (SDG 3) focuses on improving chemical regulation and minimizing workers’ exposure to hazardous chemicals;

Decent work and economic growth (SDG 8) highlights the need to protect labor rights and promote healthy and safe working environments;

Industry and innovation (SDG 9) proposes the modernization and sustainability of infrastructure and production processes, with the aim of reducing occupational hazards and enhancing workers’ health.

SDGs interact with and complement several international agendas, protocols, and agreements ratified by Brazil, with the aim of promoting health and healthy environments, including the workplace. Notable among these are the Stockholm Convention on persistent organic pollutants and the Minamata Convention on mercury. These initiatives have a significant impact on the health of workers, who are often the first victims and most affected by adverse health effects. However, the full implementation of these agendas in Brazil faces obstacles, which hinders progress toward sustainable development, comprehensive worker protection, and effective health promotion.

Achieving a balance between environmental sustainability and decent work requires several changes. These include improving and integrating health surveillance and care, especially the environmental and occupational health surveillance services, strengthening the monitoring and control of occupational hazards and exposures, and consistently promoting decent work and environmental sustainability as fundamental human rights. It is crucial to educate workers and their representatives about risks, protection, health, and rights, ensuring spaces for social participation as well as encouraging and empowering workers to engage in decision-making and defend their rights. It is also essential to translate scientific knowledge into practical applications and invest in the restructuring and strengthening of the services provided through the Brazilian Unified Health System (Sistema Único de Saúde [SUS]), encouraging comprehensive approaches that consider social determinants, with a special focus on the environment and work.

Occupational health (OH) research and actions have traditionally focused on occupational hazards and work-related diseases, often neglecting the impact of socio-environmental conditions on health disparities. Conversely, environmental health (EH) research and strategies do not always consider potential exposures and risks to workers. There is a tendency to act in silos, with little interaction between OH and EH, although both fields share common concerns and objectives in research, public policy, health surveillance, and care services.

EH discussions rarely draw attention to worker exposure in contaminated sites or following chemical accidents. For example, a study analyzing the levels of perand polyfluoroalkyl substances (PFAS) in firefighting foams used to extinguish a major fire at the Santos petrochemical terminal identified high concentrations in the marine estuary, including components banned by the Stockholm Convention.^26^ Although the study highlighted risks to the environment and exposed animals, these alarming results were not sufficient to trigger health care, prevention, or risk mitigation actions for potentially exposed workers, such as firefighters, port workers, and fishermen.

OH discussions, in turn, appear to overlook environmental exposures, domestic conditions, lifestyle choices, and social habits that can influence workers’ health, productivity, and well-being. It is essential to recognize that an individual’s biological responses depend on factors to be considered within three domains: 1) general external environment (e.g., climate, presence of green spaces, built environment, and place of residence); 2) specific external environment (e.g., lifestyle, diet, occupational hazards, exposure to cleaning products, and cosmetics); and 3) internal environment (e.g., health status, metabolic factors, microbiome, comorbidities, and inflammation). However, these factors should not be viewed separately and the integration between EH and OH is crucial to ensure that knowledge and interventions in health, environment, and work collectively benefit both individuals and communities.^27^

Parental work is often overlooked in maternal and child health studies, even when investigating respiratory, allergic, neurological, and endocrine conditions, or even cancer, which may be directly linked to parental exposure to chemical substances, such as pesticides, metals, or asbestos. These para-occupational hazards and exposures are also underexplored in OH and EH research and strategies. In situations such as the investigation of outbreaks of COVID-19 or other diseases in specific environments, such as meatpacking plants, collaboration between OH and EH could improve the investigation and mitigation of harm. Furthermore, informal home-based work involving the use of chemicals and metals, such as informal, outsourced, home-based jewelry production or gold welding processes, continuously exposes both adults and children within shared living and working spaces.^28^ Typically, OH focuses on adult health and their exposures, while EH focuses on environmental contamination and the health of populations in areas close to contaminant sources.

Despite existing challenges, EH and OH policies within the SUS underscore the interconnectedness of these fields and the importance of collaborative and coordinated efforts. The SUS Organic Law (8,080/1990) and the National Health Surveillance Policy (Política Nacional de Vigilância em Saúde [PNVS]) recognize EH and OH as essential for the promotion of comprehensive health, emphasizing the impact of social and environmental determinants on health. These policies outline specific actions in both EH and OH, assigning the responsibility for workplaces to EH. OH strategies, such as the National Workers’ Health Policy (Política Nacional de Saúde do Trabalhador e da Trabalhadora [PNSTT]) and the National Network for Comprehensive Workers’ Health Care (Rede Nacional de Atenção Integral à Saúde dos Trabalhadores [Renast]), reinforce the importance of social and environmental health determinants and the need for planning and implementing health initiatives through intraand inter-sectoral cooperation.

In Brazil, labor laws and occupational safety and health regulations have advanced significantly since the 1943 Consolidation of Labor Laws and with the progressive establishment of Regulatory Standards (Normas Regulamentadoras [NRs]), which define the obligations, rights, and duties of both employers and workers, aiming to ensure safe working environments and prevent occupational diseases and accidents. Since the publication of the first NRs in 1978, continuous NR developments and updates have been necessary to respond to the needs of occupations and economic activities and to strive to incorporate the latest scientific knowledge of occupational hazards, their health effects, and the transformations within the world of work. Some NRs provide procedures for assessment and intervention in situations of exposure to physical agents (e.g., vibration, heat, cold, humidity, noise, and radiation), chemical agents (e.g., pesticides, metals, mineral dust, benzene, and gases), and biological agents. For specific hazards, the NRs establish exposure limits, define prevention and control measures, and determine additional hazard and clinical monitoring and follow-up.^29^ However, these policies, standards, and strategies are limited, and the existing ones still need to be effectively implemented. Moreover, further progress is necessary to integrate these concepts and to align OH and EH agendas to fully embrace this transversal and collaborative vision.

To address these complex challenges, it is necessary to overcome barriers between specific areas, promoting integration between OH and EH, as well as with related fields, such as epidemiology, basic sciences, social sciences, data science, innovation, and technology. Each area can contribute unique expertise and skills, enriching collaborative efforts. Work and environment are crucial determinants of health inequities. Furthermore, it is essential to adopt innovative approaches to collect, monitor, analyze, and integrate data on exposures and health effects, aiming to protect the population against environmental and occupational hazards, prevent diseases, and promote decent, healthy, and sustainable work practices. It is also necessary to strengthen monitoring and inspections in work environments and processes and to establish continuous public health programs.

Promoting an inclusive and equitable labor market for everyone is essential, particularly for the most vulnerable groups, such as people with disabilities, migrants, Black and Mixed-race individuals, women, the LGBTQIA+ community, Indigenous peoples, *quilombola* communities, and other traditional communities. The SUS, as a universal health care system, must guarantee the health of the entire population, with special attention to the most vulnerable, prioritizing comprehensive care and health equity.

While NRs are quite broad in addressing several environmental risk factors, they face certain limitations in ensuring comprehensive health for all workers. For example, the quantitative assessment of occupational heat exposure considers a worker’s heat overload and metabolic rate. However, it specifically focuses on exposure to heat in enclosed environments or from artificial heat sources, neglecting frequent exposures such as those faced by agricultural workers, construction workers, urban cleaners, and wildland firefighters. Another important limitation is that the NRs only cover formally employed workers, i.e., those hired with a signed employment contract, leaving millions of informal workers unprotected - approximately 41% of workers in Brazil - who are potentially highly vulnerable and exposed to occupational hazards.

Considering that the exposure limits recommended by the NRs aim to protect formal workers, it is crucial that the same safety standards, including prevention and control measures and workplace inspections, are extended to informal workers. This can be achieved through coordinated actions among different branches of the Brazilian Ministry of Health, Ministry of Labor, and Ministry of the Environment, among others, in order to safeguard public health and prevent increased patient care costs. Therefore, expanding the scope of these NRs to encompass all workers, especially considering the universality of the SUS, is imperative for protecting the health and safety of the entire population.

Successful interventions to tackle climate change among workers include the implementation of a water-rest-shade program for farmers exposed to extreme heat in Central America.^30^ In Brazil, these prevention and intervention actions - although urgent - are still in their early stages and need to be integrated and coordinated across all levels of care and services within the SUS, paying special attention to the most vulnerable populations and workers and considering all potential effects of environmental changes on workers.

### FINAL REFLECTIONS

Work and the environment are key determinants of the health-disease process, and their recent transformations - and deteriorations - have led to inestimable human, social, environmental, and financial burdens on society, requiring ongoing discussion and action. To reduce health disparities, promote environmental balance, and ensure decent work, it is necessary to recognize the inseparable relationship between work, environment, and health and to improve the effectiveness and sustainability of the measures and policies adopted.

The SDGs, along with other local, national, and international agendas, provide pathways and guidelines for promoting safe and healthy environments, including the workplace. However, effective implementation of these guidelines still faces major obstacles. Therefore, it is vital to prioritize integrated strategies that improve both environmental and working conditions for the population in the EH and OH agendas. Establishing inclusive and equitable policies and strategies, mitigating environmental and occupational hazards, and protecting the most vulnerable are essential measures to reduce health disparities and promote social, environmental, and labor justice, especially in lower-income countries.
